# Clinical Outcomes of Transcatheter Versus Surgical Aortic Valve Replacement in Patients with Pure Native Aortic Regurgitation: A Systematic Review and Meta-Analysis

**DOI:** 10.3390/jcdd13100486

**Published:** 2026-09-26

**Authors:** Honghong Ke, Shirong Wang, Jinyi Li, Guoqiang Zhong

**Affiliations:** 1 Department of Cardiology, The First Affiliated Hospital of Guangxi Medical University, Nanning 530021, China; khhred2012@126.com (H.K.); 13978877651@163.com (S.W.); 2 Department of Cardiology, The First Affiliated Hospital of Guilin Medical University, Guilin 541001, China; yzrcg@126.com

**Keywords:** aortic valve regurgitation, transcatheter aortic valve replacement, surgical aortic valve replacement

## Abstract

Background: Aortic valve regurgitation is a common valvular heart disease. Transcatheter aortic valve replacement (TAVR) and surgical aortic valve replacement (SAVR) are the principal therapeutic modalities, while the comparative efficacy of these two approaches remains controversial. Methods: This systematic review and meta-analysis was prospectively registered on PROSPERO (registration number: CRD420261411339). We systematically searched Web of Science, PubMed, EMBASE, and the Cochrane Library databases for relevant studies from their inception to 26 July 2026. RevMan 5.4 software was used to conduct this analysis. Odds ratios (OR) with 95% confidence intervals (CI) were used to evaluate the outcomes. Results: We assessed in-hospital outcomes and post-discharge 30-day outcomes. The primary outcomes were length of hospital stay, perioperative complications, and valve condition. Secondary outcomes included all-cause mortality, cardiovascular mortality, new-onset atrial fibrillation (AF), and new permanent pacemaker implantation (PPI). Nine articles involving 28,811 patients were included. Compared with SAVR, TAVR reduced length of hospital stay [mean difference (MD) = −4.49, 95% confidence interval (CI): −4.98, −4.01, *p* < 0.00001, I2 = 80%], perioperative complications [Odds ratios (OR): 0.55, 95% CI: 0.50 to 0.60, *p* < 0.00001, I2 = 62%) and new-AF(OR: 0.50, 95% CI: 0.43 to 0.59, *p* < 0.00001 I2 = 30%). However, there was a significant increase in perivalvular leakage (OR: 17.13, 95% CI: 4.13 to 71.13, *p* < 0.0001 I2 = 29%) and new PPI (OR: 2.63, 95% CI: 2.13 to 3.25, *p* < 0.00001 I2 = 80%). There was no significant difference observed in all-cause mortality (OR: 0.91, 95% CI: 0.69–1.20, *p* = 0.49, I2 = 69%). Conclusions: Compared with SAVR, TAVR reduced length of hospital stay, perioperative complications, and new AF. However, it was associated with an increase in perivalvular leakage and new PPI. These findings suggest that TAVR may represent a better strategy for patients with pure native aortic regurgitation.

## 1. Introduction

Aortic valve regurgitation (AR) is a valvular heart disease resulting from aortic valve insufficiency, thereby causing volume overload in the left ventricle. Its pathophysiological mechanism is characterized by the retrograde flow of blood from the aorta into the left ventricle during diastole. Progressively, this can result in compensatory left ventricular dilation and hypertrophy, eventually progressing to heart failure [1]. Traditionally, surgical aortic valve replacement (SAVR) has been the gold standard for treating severe AR, especially for young patients with low surgical risk, demonstrating established efficacy and a favorable long-term prognosis. A retrospective study of SAVR in patients with pure severe AR showed that among 112 patients, the in-hospital mortality rate was 2.7%, the 30-day mortality rate was 3.6%, and postoperative echocardiography demonstrated a significant reduction in AR [2]. However, SAVR requires extracorporeal circulation and sternotomy, entailing substantial surgical trauma. In high-risk patients, such as the elderly, those with multiple comorbidities, or those with severe left ventricular dysfunction, surgical tolerance is limited, and perioperative complications and mortality rates are significantly increased. Therefore, identifying a less invasive alternative treatment option has become a pressing clinical priority.

Since the first successful implementation of transcatheter aortic valve replacement (TAVR) in 2002, it was initially indicated for patients with severe aortic valve stenosis (AS) who were at high surgical risk or were unsuitable for surgery [3]. Subsequent studies have confirmed its safety and efficacy [4]. Multiple randomized controlled trials and meta-analyses have demonstrated that in patients with AS, TAVR is superior to SAVR with respect to short-term mortality and is associated with a lower incidence of complications such as bleeding and acute kidney injury [5,6]. In recent years, with advancements in equipment and the accumulation of operational experience, researchers have begun to explore its application in pure AR therapy. However, unlike AS, the complex anatomy in patients with pure AR presents a primary challenge for TAVR. Due to the absence of the common valve leaflet calcification seen in AS patients, the TAVR valve fails to secure sufficient radial support force for anchoring, increasing the risk of valve displacement or embolism [7]. Furthermore, AR patients often exhibit significant dilation of the aortic valve ring and the left ventricular outflow tract, which further complicates the selection of valve size [8]. Moreover, inadequate coaptation of the valve leaflets in AR patients leads to an increased risk of residual regurgitation after valve deployment [9]. One study compared the clinical efficacy of TAVR and SAVR for the treatment of high-risk severe AR. The results showed that the TAVR group had a shorter operation time, less intraoperative blood loss, and a shorter hospital stay compared to the SAVR group, with no increase in the all-cause mortality rate [10]. A further study utilizing data from the United States healthcare database indicates that the mid-term mortality rate of TAVI in patients with pure aortic regurgitation is higher than that associated with SAVR [11]. These discrepant findings underscore the need for comprehensive comparative studies. Notably, existing comparative studies for pure native AR are limited by substantial clinical and methodological heterogeneity. Patient cohorts differ widely in age, surgical risk profile, left-ventricular remodeling status, and comorbidity burden. Moreover, TAVR devices include both general-purpose aortic valves and AR-dedicated prostheses, while procedural access routes (transapical versus transfemoral) vary across centers. Furthermore, most available evidence originates from retrospective registries rather than randomized controlled trials, characterized by inconsistent definitions for perioperative adverse events. These heterogeneities contribute to conflicting conclusions among published reports and reinforce the need for a contemporary systematic meta-analysis synthesizing currently available data.

This study aims to rigorously evaluate the existing literature, conduct a quantitative synthesis of data from high-quality studies, and compare TAVR and SAVR regarding perioperative and 30-day post-discharge length of hospital stay, perioperative complications, valve condition, all-cause mortality, cardiovascular mortality, new-onset AF, and new PPI in adult patients with Pure AR. The findings of this study are expected to provide a critical evidence base for clinical decision-making in optimizing the selection of surgical approach for pure AR patients.

## 2. Data Sources

A systematic search for eligible studies was conducted across the Web of Science, PubMed, EMBASE, and Cochrane Library databases, covering publications up to July 26, 2026. A manual reference search was conducted to identify studies meeting the inclusion criteria and strengthen the robustness of the study’s conclusions. The search terms include: transcatheter aortic valve replacement, TAVR, surgical aortic valve replacement, SAVR, pure aortic regurgitation. The keywords were combined using Boolean logical operators (AND, OR) to comprehensively obtain studies comparing TAVR and SAVR. The search strategy used in this study is outlined in Table 1. The reporting of the study followed the Preferred Reporting Items for Systematic Reviews and Meta-Analyses (PRISMA). The completed PRISMA 2020 checklist is provided as Supplementary Material S1. This systematic review and meta-analysis was prospectively registered on PROSPERO (registration number: CRD420261411339). The review protocol was written prior to literature screening.

### 2.1. Eligibility Criteria

Criteria for inclusion: (1) Adult patients (aged ≥18 years) with AR who are receiving TAVR treatment or SAVR treatment; (2) The study must report the in-hospital outcomes and post-discharge 30-day outcomes; (3) Randomized controlled trial (RCT), cohort study, or retrospective study.

Exclusion criteria were as follows: (1) Animal studies, in vitro studies, reviews, meta-analyses, duplicate publications, editorials, comments, abstracts of conferences without full text, or qualitative research; (2) Studies that do not have available or cannot extract data on in-hospital outcomes and post-discharge 30-day outcomes.; (3) The baseline population includes participants who have AS.

### 2.2. Selection of Studies, Data Extraction, and Management

Two researchers conducted the literature search and data extraction independently. Disagreements were resolved through consultation with a third researcher. Data extracted from studies included study characteristics, patient characteristics, in-hospital outcomes, and post-discharge 30-day outcomes. The primary outcomes were length of hospital stay, perioperative complications, and valve condition. Secondary outcomes included all-cause mortality, cardiovascular mortality, new-onset AF, and new PPI.

### 2.3. Certainty of Evidence

Since all nine included studies were cohort studies, we strictly applied the Newcastle-Ottawa Scale (NOS) version tailored for this study design. This scale evaluates three domains: selection of study groups, comparability of groups, and ascertainment of the outcome of interest. Each study could receive a maximum of nine stars. Two investigators independently performed the quality assessment; discrepancies were resolved via discussion or consultation with a third investigator.

### 2.4. Data Synthesis and Analysis

Revman5.4 software (Cochrane Collaboration, Oxford, UK) was used to comprehensive quantitative analysis. Odds ratios (OR) with 95% confidence intervals (CIs) were used to assess the outcome. A *p* value ≤0.05 was considered statistically significant whereas a *p* value > 0.05 was considered insignificant. The study utilized Q-value statistic tests and I2 tests to assess heterogeneity among studies. If the *p* value of Cochran’s Q test was <0.10 and I2 was >50%, there was a heterogeneity in the study. The effect model depended on heterogeneity levels. If I2 was ≥50%, a random-effects statistical model was used during analysis, and if I2 was <50%, a fixed-effect model was used. Sensitivity analysis was conducted using the leave-one-out approach, and publication bias was visually evaluated via a funnel plot generated using standard statistical software.

## 3. Results

### 3.1. Characteristics of the Included Studies

The literature search yielded a total of 1150 publications. A total of nine studies (enrolled between 2019 and 2026) were ultimately included in this meta-analysis, in accordance with the prespecified inclusion and exclusion criteria [11,12,13,14,15,16,17,18,19]. Figure 1 illustrates the literature screening process.

Mostly derived from retrospective cohort studies, a total of 28,811 participants with AR were included in this analysis, of whom 4854 were assigned to TAVR and 23,957 to SAVR, as shown in Table 2. All included studies were non-randomized cohort studies. Assignment to TAVR or SAVR was determined by local heart team decisions. Common clinical considerations included age, surgical risk scores, annular anatomy, presence of valve calcification, comorbid burden, frailty, and local device availability. Some studies applied propensity score matching to balance baseline covariates; however, unmeasured confounders could not be fully eliminated.

The baseline features of the participants have been listed in Table 3. The ages ranged from 58.7 ± 14.5 to 75.81 ± 9.40 years. The mean percentages of participants by sex, heart failure, hypertension, diabetes, coronary artery disease, cerebral vascular disease, peripheral vascular disease, chronic obstructive pulmonary disease (COPD), AF, and a permanent pacemaker have also been listed.

Table 4 presents the echocardiography characteristics of patients before operation.

### 3.2. Quality Evaluation and Risk of Bias

All nine included studies were cohort studies (retrospective single-center cohorts or nationwide registry cohorts); no case-control studies were identified following the screening process. Quality assessment was performed using the cohort-specific NOS. Individual NOS star ratings for each study are detailed in Table 5.

### 3.3. Outcome Measures

In total, 3935 patients in the TAVR group versus 13,139 patients in the SAVR group completed the hospital stay day (Figure 2, MD = −4.49, 95% CI [−4.98, −4.01], I2 = 80%, *p* < 0.00001). 53 patients in the TAVR group versus 49 patients in the SAVR group completed the intensive care unit (ICU) stay day (Figure 2, MD = −0.05, 95% CI [−0.86, 0.76], I2 = 0%, *p* < 0.00001). The results showed that TAVR treatment was more effective in reducing the hospital stay days and ICU stay days compared with SAVR, with a statistically significant difference observed. To address the substantial heterogeneity, we performed a leave-one-out sensitivity analysis by sequentially excluding each of the four studies. When the Zhou2023 study [19] was excluded, the heterogeneity was reduced from 80% to 13%.

Perioperative complications encompassed stroke/TIA, AKI, major bleeding, major vascular complications, postoperative severe heart failure, and postoperative infection. As illustrated in Figure 3, pooled analyses revealed that transcatheter aortic valve replacement (TAVR) was associated with significantly lower risks of stroke/TIA (odds ratio [OR] = 0.55; 95% confidence interval [CI]: 0.42–0.73; *p* < 0.0001; I2 = 28%), AKI (OR = 0.62; 95% CI: 0.55–0.70; *p* < 0.00001; I2 = 78%), major bleeding (OR = 0.30; 95% CI: 0.23–0.40; *p* < 0.00001; I2 = 0%), severe postoperative heart failure (OR = 0.65; 95% CI: 0.43–0.97; *p* = 0.04; I2 = 46%), and postoperative infection (OR = 0.42; 95% CI: 0.28–0.62; *p* < 0.0001; I2 = 0%). In contrast, TAVR was associated with a significantly higher risk of major vascular complications (OR = 2.45; 95% CI: 1.30–4.61; *p* = 0.006; I2 = 54%). Collectively, these findings indicate that TAVR confers a more favorable perioperative safety profile with respect to stroke/TIA, AKI, major bleeding, severe postoperative heart failure, and postoperative infection. However, it carries an elevated risk of access-related major vascular complications.

Subsequently, valve-related outcomes were compared between the TAVR and SAVR groups, as illustrated in Figure 4. Pooled analyses revealed that TAVR was associated with a significantly higher risk of perivalvular leakage (OR = 17.13; 95% CI: 4.13–71.13; *p* < 0.0001), with low interstudy heterogeneity (I2 = 29%). For valve embolization and the need for a second valve implantation, the pooled OR was 7.92 (95% CI: 0.89–70.08; *p* = 0.06), indicating no statistically significant difference between the TAVR and SAVR groups; no heterogeneity was observed (I2 = 0%). The composite endpoint of device-related complications, encompassing both perivalvular leakage and valve embolization/second valve implantation, demonstrated a substantially elevated risk with TAVR (OR = 14.20; 95% CI: 4.34–46.43; *p* < 0.0001). Formal subgroup analysis showed no statistically significant difference between these two component endpoints (*p* = 0.56).

As illustrated in Figure 5, pooled analyses revealed no statistically significant difference in all-cause mortality between TAVR and SAVR (OR = 0.91; 95% CI: 0.69–1.20; *p* = 0.49), although substantial interstudy heterogeneity was observed (I^2^ = 69%). Similarly, cardiovascular mortality did not differ significantly between the two groups (OR = 1.63; 95% CI: 0.55–4.81; *p* = 0.37), with no evidence of heterogeneity (I^2^ = 0%). A composite analysis of all mortality endpoints, including both all-cause and cardiovascular mortality, yielded an overall OR of 0.94 (95% CI: 0.72–1.23; *p* = 0.65). Formal testing for subgroup differences confirmed the absence of a statistically significant divergence between all-cause and cardiovascular mortality estimates (*p* = 0.30).

As shown in Figure 6. The pooled fixed-effect meta-analysis demonstrated that patients undergoing TAVR had a significantly lower risk of new-onset AF (OR = 0.50, 95% CI: 0.43–0.59, *p* < 0.00001). Low between-study heterogeneity was observed (I^2^ = 30%).

As shown in Figure 7. The pooled meta-analysis revealed that the risk of new PPI was significantly higher in the TAVR cohort (OR = 2.63; 95% CI: 2.13–3.25; *p* < 0.00001). Substantial inter-study heterogeneity was observed (I^2^ = 80%). To address the substantial heterogeneity, we performed a leave-one-out sensitivity analysis by excluding each of the eight studies sequentially. When the Alharbi2020 study [18] was excluded, the heterogeneity was reduced from 80% to 0%.

## 4. Discussion

This study systematically evaluated the efficacy and safety of TAVR and SAVR in patients with pure AR. The main findings are as follows: 1. Regarding short-term mortality, there was no significant difference between TAVR and SAVR, suggesting that TAVR demonstrates an acceptable safety profile in patients with isolated AR. However, given the observational study design and limited follow-up duration, this finding cannot be extrapolated to long-term survival. 2. The incidence of perioperative complications (including stroke/TIA, AKI, major bleeding, major vascular complications, postoperative severe heart failure, and postoperative infection) in the TAVR group was significantly lower than that in the SAVR group; additionally, hospital stays were significantly shorter, and the incidence of new-onset atrial fibrillation was significantly reduced, thereby demonstrating the minimally invasive advantages of TAVR. These findings are largely explained by its minimally invasive nature, which avoids sternotomy and cardiopulmonary bypass. Avoidance of open chest surgery reduces surgical trauma, systemic inflammatory response, bleeding risk, and postoperative infection risk. The lower incidence of new-onset atrial fibrillation may also be secondary to less surgical myocardial and pericardial manipulation. These advantages are clinically meaningful, especially for elderly or comorbidity-burdened patients, who may benefit from faster post-procedural recovery. Nevertheless, it should be noted that TAVR carried a higher risk of major vascular access-related complications, which represents a procedure-specific safety concern requiring careful pre-procedural vascular screening. 3. The permanent pacemaker implantation rate and the incidence of paravalvular leakage in the TAVR group were significantly higher than those in the SAVR group, representing the primary technical challenges faced by TAVR in patients with AR. The significantly elevated risk of perivalvular leakage may be attributed to the absence of native valve calcification, which eliminates the calcified substrate for stable anchoring and sealing of transcatheter prostheses. The aortic annulus and left ventricular outflow tract dilation further exacerbate prosthesis annulus mismatch. Higher permanent pacemaker implantation rates reflect injury to the atrioventricular conduction system during prosthesis deployment. From a clinical perspective, perivalvular leakage may worsen ventricular volume overload and negatively impact long-term prognosis, while de novo permanent pacing carries risks of pacemaker-related complications. These technical limitations highlight that patient-specific anatomical screening and selection of AR-dedicated valve devices are critical prerequisites for TAVR in pure AR. All comparative endpoints should be evaluated concurrently, rather than emphasizing the benefits of a single intervention in isolation. While TAVR offers short-term advantages, including minimally invasive access and rapid recovery, these must be weighed against its higher incidence of perivalvular regurgitation, new PPI, and major vascular complications. Of particular clinical significance are perivalvular regurgitation and new PPI, as both have been associated with adverse long-term outcomes. Notably, although short-term all-cause and cardiovascular mortality were comparable between treatment groups in this analysis, these device-related complications may compromise durability and functional status over time.

In the statistical analysis of the length of hospital stay and the new PPI, we observed a high degree of heterogeneity. When we performed a leave-one-out sensitivity analysis by excluding the Zhou 2023 study [19] or the Alharbi 2020 study [18], the overall heterogeneity significantly decreased. From a methodological standpoint, this finding suggests that the Zhou 2023 study [19] or the Alharbi 2020 study [18] may have introduced a systematic difference, such as unique patient selection criteria, a different procedural technique, or a distinct outcome assessment method.

The anatomical characteristics of patients with pure AR represent the primary technical challenge for TAVR. Unlike patients with aortic stenosis (AS), patients with pure AR usually lack valve calcification, which results in insufficient anchoring of the valve stent and increases the risk of valve displacement or recurrence of regurgitation [20,21]. Due to the lack of calcified anchoring structures, the stability of traditional transcatheter heart valves (THV) when deployed among patients with pure AR is significantly reduced. The failure rate of early TAVI devices (such as the first-generation balloon valvuloplasty) historically reached 10–15%, severely hindering the widespread adoption of this technology [7,22]. Furthermore, aortic root dilation and valve annulus asymmetry pose significant challenges for valve size selection. The surgical procedure may injure the conduction system, resulting in a higher incidence of permanent pacemaker implantation [23,24]. To address the absence of calcified anchor points in the anatomy of patients with pure AR, the development of new valve systems and the optimization of procedural strategies are key to improving the success rate of TAVR. Therefore, a valve system specifically designed for AR has emerged. Notably, the Jena-Valve Trilogy system, through its unique “locking key” design, clamps directly onto the native valve leaflets, achieving stable anchoring and significantly improving the surgical success rate [25]. Clinical studies indicate that TAVR performed with specialized devices (Jena-Valve and J-Valve) yields 30-day and 1-year mortality rates of 4.7% and 8.7%, respectively, which are significantly lower than those observed in patients treated with first-generation (4.7% and 27.2%) or second-generation (7.2% and 12.7%) non-specialized valves [26]. In addition, the new generation of self-expanding valves, such as Evolut PRO and ACURATE neo, enhances radial support force and sealing performance within non-calcified annuli through improved valve frame design and outer skirt structure, thereby reducing the incidence of paravalvular leakage [27]. The results of this study align closely with previous research. Numerous studies have demonstrated that technological advancements and the introduction of novel valve systems have progressively enhanced the efficacy of TAVR in patients with AR. Notably, valve systems such as the J-Valve, which are specifically designed for AR, effectively mitigate challenges related to valve positioning and fixation through a unique anchoring mechanism, significantly reducing the incidence of paravalvular leakage. However, conduction system damage remains the primary complication of TAVR and necessitates further technical refinement. Taken together, these technical considerations underline the importance of careful pre-procedural anatomical evaluation when selecting candidates for TAVR in pure native AR.

## 5. Limitations of This Study

This study has the following limitations: 1. All the included studies are retrospective observational studies, which are susceptible to selection bias and confounding factors. Although some studies used propensity score matching, residual confounding cannot be entirely eliminated. Treatment assignment was non-randomized: clinicians tended to allocate TAVR to older, frailer patients with higher surgical risk and SAVR to younger, low-risk individuals. Unmeasured confounders, including frailty, anatomical suitability for TAVR, and operator experience, may bias effect-size estimates. 2. Included studies pooled transfemoral and transapical TAVR participants within one TAVR group. These access routes differ substantially in baseline risk and procedural risks. Without individual-patient data, we could not perform access-site-stratified subgroup analyses, which may contribute to residual heterogeneity. 3. There is heterogeneity among the studies in terms of patient selection criteria, valve types, and surgical techniques, which may compromise the robustness of the combined results. 4. The relatively short follow-up duration precludes the assessment of long-term efficacy and valve durability. 5. The number of studies that included certain outcome indicators is relatively small, which limits the conduct of subgroup analysis and sensitivity analysis.

## 6. Conclusions

This meta-analysis of observational cohort studies in patients with pure native aortic regurgitation indicates that TAVR is associated with a shorter length of hospital stay, a lower overall perioperative complication burden, and a reduced incidence of new-onset AF compared with SAVR. Conversely, TAVR was linked to significantly higher rates of perivalvular regurgitation, new PPI, and major vascular complications related to access site management. No statistically significant differences were observed between the two groups in short-term all-cause or cardiovascular mortality. Given that all included studies were non-randomized, potential selection bias and residual confounding remain important limitations. These findings suggest that TAVR may represent a viable alternative therapeutic option for select patients. Future prospective multicenter randomized controlled trials are warranted to confirm these findings and establish optimal patient selection criteria for TAVR in patients with pure native aortic regurgitation.

## Figures and Tables

**Figure 1:**
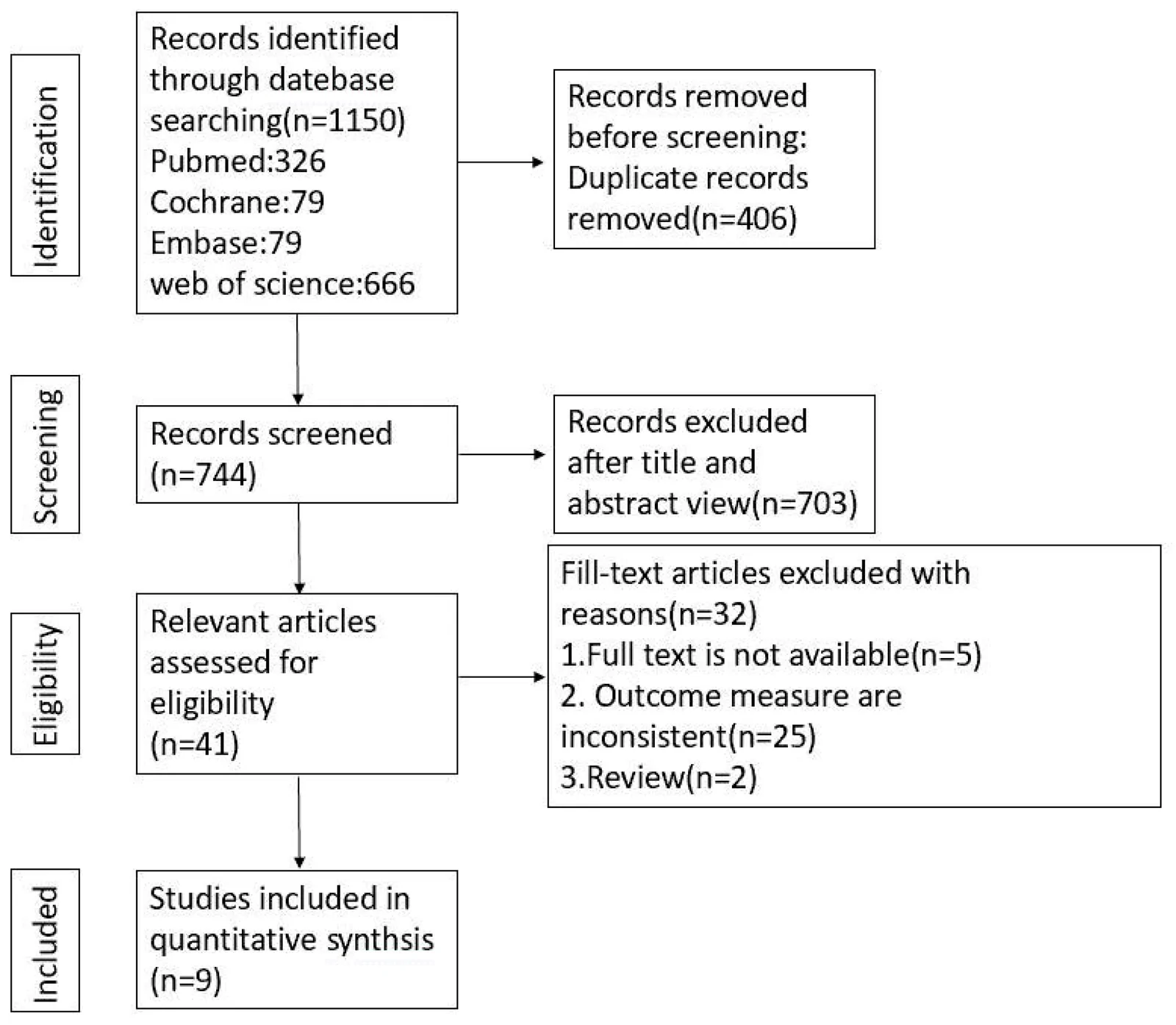
Flowchart of the literature screening.

**Figure 2:**
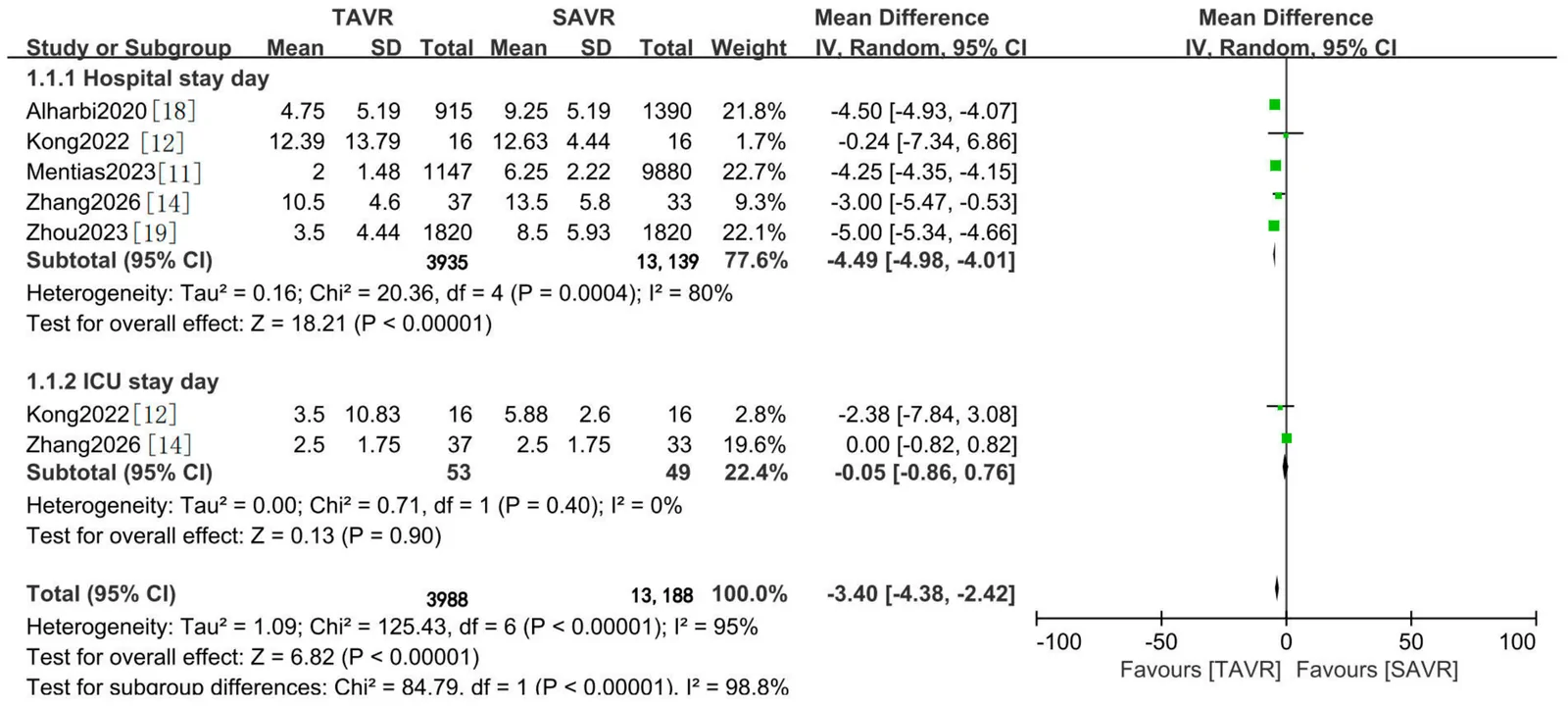
Forest plot of hospital length of stay [11,12,14,18,19].

**Figure 3:**
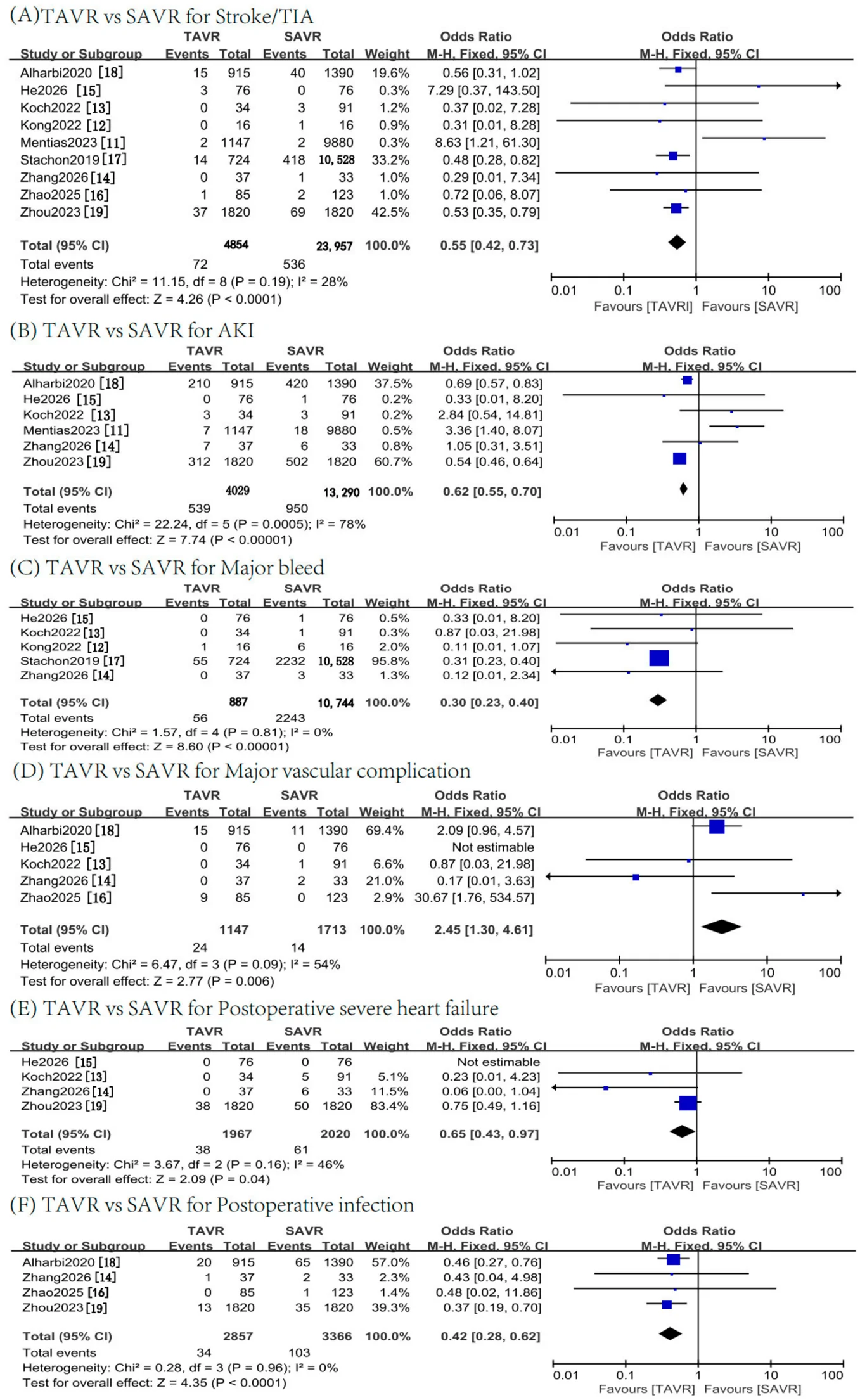
Perioperative complications [11,12,13,14,15,16,17,18,19].

**Figure 4:**
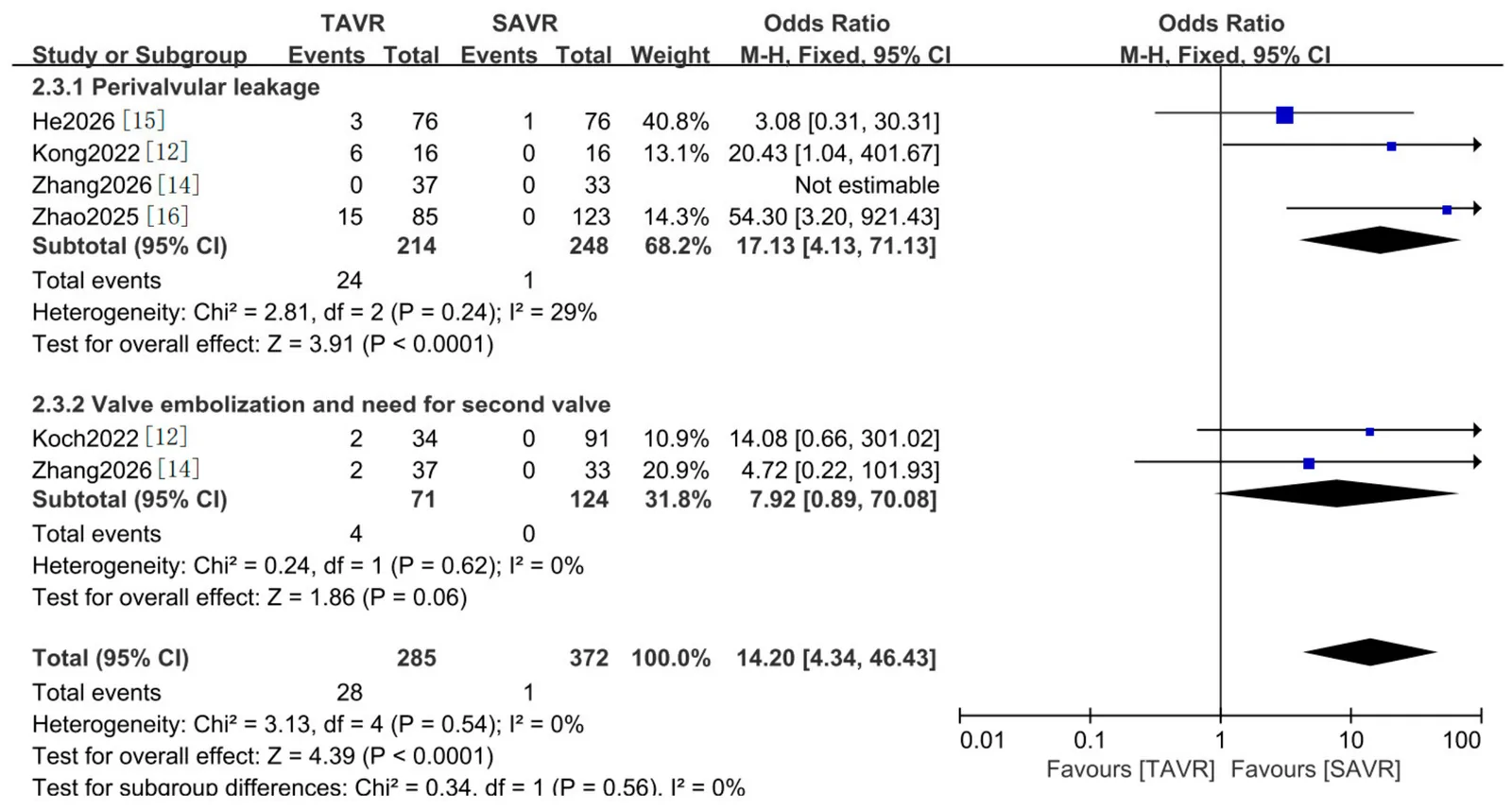
Forest plot of valve condition [12,14,15,16].

**Figure 5:**
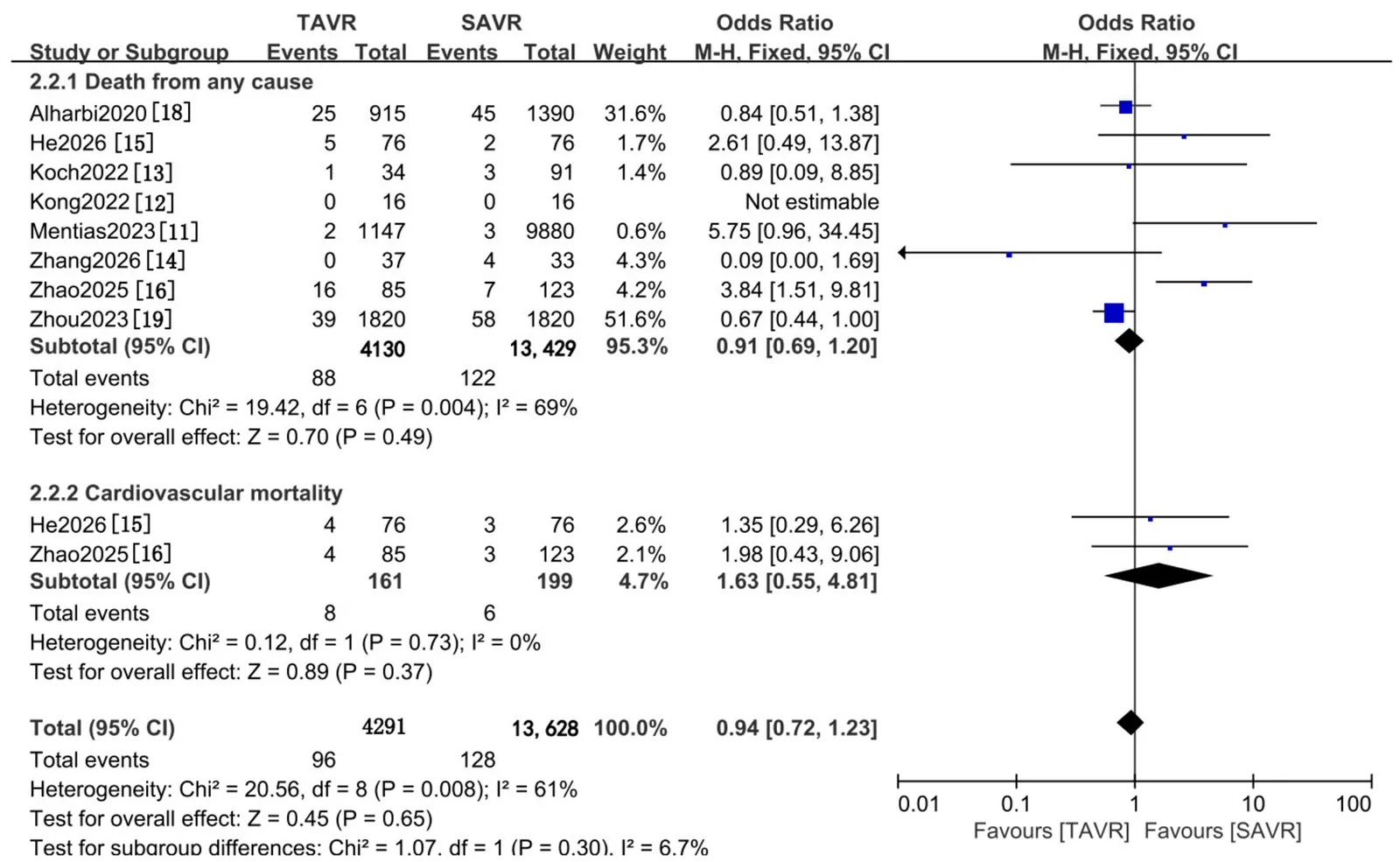
Forest plot of mortality [11,12,13,14,15,16,18,19].

**Figure 6:**
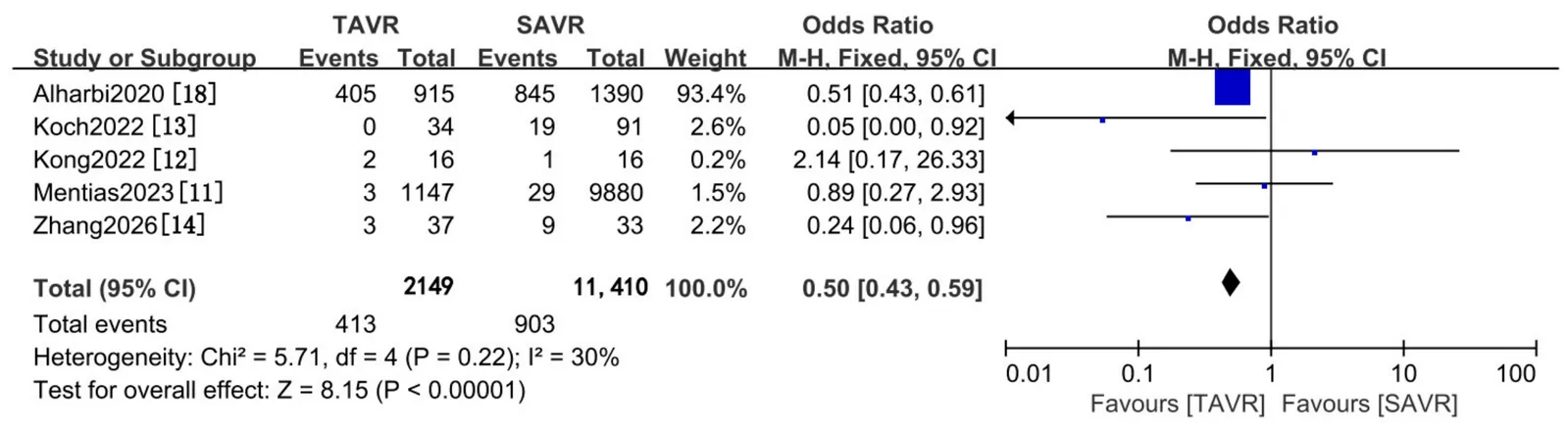
Forest plot of new-onset AF [11,12,13,14,18].

**Figure 7:**
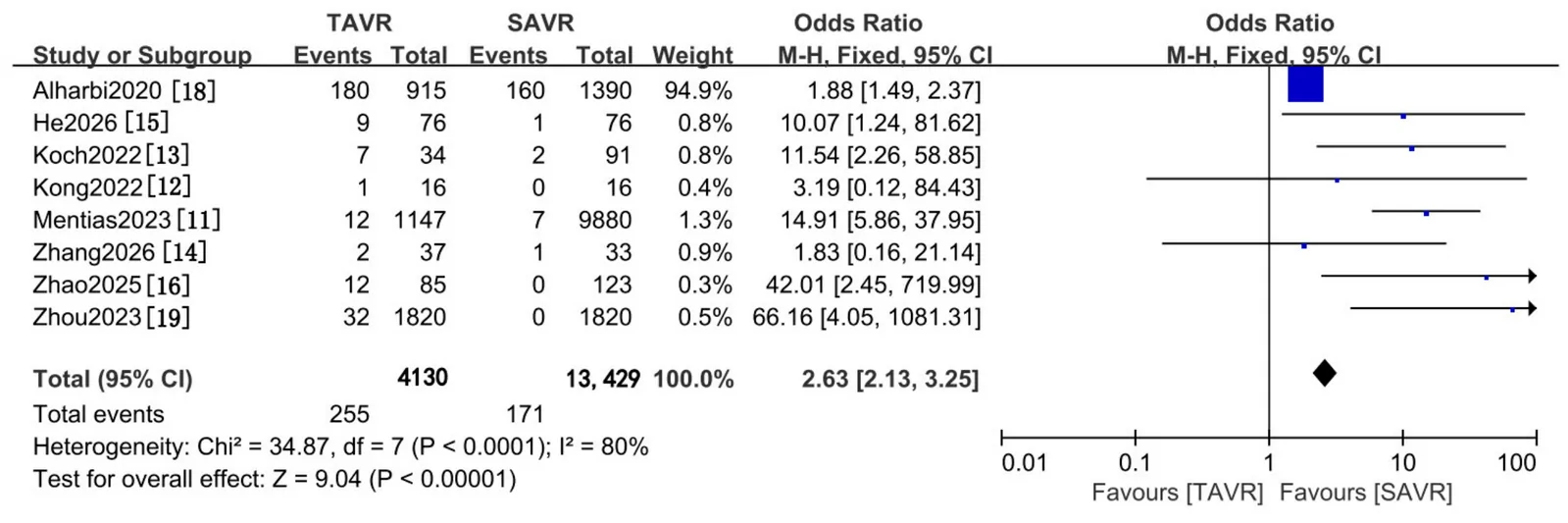
Forest plot of new PPI [11,12,13,14,15,16,18,19].

**Table 1:** Search strategy for PUBMED.

No	Search Terms
1	Transcatheter Aortic Valve Replacement.ti,mesh.
2	TAVR.ti,ab.
3	Transcatheter Aortic Valve Implantation.ti,ab
4	or 1–3
5	Surgical Aortic Valve Replacement.ti,mesh.
6	SAVR.ti,ab.
7	or 5–6
8	Aortic Valve Insufficiency.ti,mesh.
9	Insufficiency, Aortic Valve.ti,ab.
10	Aortic Incompetence.ti,ab.
11	Incompetence, Aortic.ti,ab.
12	Aortic Valve Incompetence.ti,ab.
13	Incompetence, Aortic Valve.ti,ab.
14	Regurgitation, Aortic Valve.ti,ab.
15	Aortic Regurgitation.ti,ab.
16	Regurgitation, Aortic.ti,ab.
17	or 8–16
18	4 and 7 and 17

Abbreviations: ti: title; ab: abstract.

**Table 2:** The baseline of the studies.

Study	Study Type	TAVR (n)	SAVR (n)	Time Period of Participants’ Enrollment (Years)	Valve System Configuration in TAVR	TAVR Procedure Pathway
Kong2022 [12]	RCS	16	16	2016–2021	J-valve system (100%)	Transapical (100%)
Koch2022 [13]	RCS	34	91	2014–2020	Edwards Sapien 3 (50%) CoreValve Evolut R/Pro (50%)	Transfemoral (94.1%) Transapical (5.9%)
Zhang2026 [14]	RCS	37	33	2021–2024	Self-expanding valve systems (Not specified)	Transfemoral (100%)
He2026 [15]	RCS	76	76	2018–2023	J-valve system (100%)	Transapical (100%)
Zhao2025 [16]	RCS	85	123	2018–2023	J-valve system (100%)	Transapical (100%)
Stachon2019 [17]	NCS	724	10,528	2008–2015	Not specified	Transfemoral (65.7%) Transapical (34.3%)
Alharbi2020 [18]	NCS	915	1390	2016–2017	Not specified	Not specified
Zhou2023 [19]	NCS	1820	1820	2016–2019	Not specified	Not specified
Mentias2023 [11]	RCS	1147	9880	2016–2019	Not specified	Transfemoral (100%)

Abbreviations: TAVR: Transapical Aortic Valve Replacement; SAVR: Surgical aortic valve replacement; NCS: Nationwide cohort study; RCS: Retrospective cohort study.

**Table 3:** The baseline characteristics of patients.

Study	Age, Years	Sex, Females (%)	Heart Failure (%)	Hypertension (%)	Diabetes (%)	Coronary Artery Disease (%)	Cerebral Vascular Disease (%)	Peripheral Vascular Disease (%)	COPD (%)	Atrial Fibrillation (%)	Permanent Pacemaker (%)
	TAVI/ SAVR	TAVI/ SAVR	TAVI/ SAVR	TAVI/ SAVR	TAVI/ SAVR	TAVI/ SAVR	TAVI/ SAVR	TAVI/ SAVR	TAVI/ SAVR	TAVI/ SAVR	TAVI/ SAVR
Kong 2022 [12]	68.31 ± 5.10/ 67.94 ± 5.51	18.7/ 31.2	81.3/ 68.8	75.0/ 68.8	6.3/ 12.5	50/ 31.3	12.5/ 12.5	6.3/ 6.3	12.5/ 25.0	0/ 6.3	6.3/ 6.3
Koch 2022 [13]	68 ± 12.2/ 58.7 ± 14.5	26.5/ 22.0	29.4/ 34.0	4.7/ 4.7	26.5/ 15.4	64.7/22.0	11.8/ 11.0	23.5/ 6.6	29.4/ 4.4	44.1/ 7.7	8.8/ 3.3
Zhang 2026 [14]	64 (36,78)/ 64 (39,74)	29.7/ 18.2	49/ 42	38/ 58	14/ 6	-	14/ 15	-	-	-	-
He 2026 [15]	66 (65, 68)/ 65 (61,68)	32.9/ 25.0	86.8/ 86.8	28.9/ 23.7	10.5/ 6.6	19.7/ 26.3	-	-	11.8/ 3.9	3.9/ 3.9	0/0
Zhao 2025 [16]	70.1 (66.0, 73.0)/ 70.0 (67.0, 72.0)	21.7/ 24.9		86.2/ 85.6	8.7/ 11.0	50.2/ 48.1	10.3/ 11.0	3.5/ 2.8	7.5/ 7.7	6.7/ 2.2	1.2/0
Stachon2019 [17]	75.81 ± 9.40/ 60.9 ± 14.1	39.9/ 26.0	46.0/ 25.2	65.0/ 56.8	17.5/ 12.2	41.5/ 10.1	3.5/ 1.5	14.1/ 3.0	16.1/ 7.9	44.3/ 35.7	-
Alharbi2020 [18]	78 (68–85)/ 74 (67–80)	265 (29.0)/400 (28.8)	79.8/ 76.3	44.8/ 40.3	27.4/ 23.0	62.8/ 59.7	8.2/ 9.4	25.2/ 21.9	25.1/ 23.0	-	-
Zhou 2023 [19]	70.49 ± 12.49/ 69.98 ± 10.41	36.0/ 35.7	73.3/ 72.9	-	15.8/ 15.5	35.1/ 36.3	8.4/ 8.7	25.5/ 24.1	26.7/ 26.9	-	9.7/ 10.2
Mentias2023 [11]	75.6 ± 6.8/ 75.6 ± 5.7	40/ 40	64/ 64	85/ 85	27/ 27	60/ 60	8/ 8	26/ 26	-	28/ 28	7/ 7

COPD: Chronic Obstructive Pulmonary Disease.

**Table 4:** Echocardiography characteristics of patients.

Study	LVEF (%)	MR ≥ Moderate (%)	TR ≥ Moderate (%)	LVIDd (cm)	LVIDs (cm)
	TAVI/ SAVR	TAVI/ SAVR	TAVI/ SAVR	TAVI/ SAVR	TAVI/ SAVR
Kong2022 [12]	55.21 ± 13.17/ 57.54 ± 12.07				
Koch2022 [13]	50.2 ± 13.7/ 51.4 ± 10.0	37.5/6.9	21.9/10.3	5.6 ± 0.9/ 5.9 ± 0.8	4.2 ± 1.0/ 4.4 ± 0.9
Zhang2026 [14]	63 (35–74)/ 62 (39–75)				
He2026 [15]	59 (50, 64)/ 60 (54, 65)			57.0 (52.0, 63.0)/ 57.0 (52.0, 61.5)	38.3 (34.8,47.6)/ 38.8 (34.2,44.5)
Zhao2025 [16]	57.7 (53.0, 65.0)/ 57.5 (54.7, 63.0)	23.6/8.5	12.6/3.4	58.0 (52.0, 61.0)/ 59.3 (55.0, 63.0)	39.9 (33.0,45.0)/ 40.3 (35.0,44.3)
Stachon2019 [17]					
Alharbi2020 [18]					
Zhou2023 [19]					
Mentias2023 [11]					

LVEF, Left Ventricular Ejection Fraction; LVIDd, Left Ventricular Internal Diameter end Diastole; LVIDs, Left Ventricular Internal Diameter end Systole; MR, Mitral Re-gurgitation; TR, Tricuspid Regurgitation.

**Table 5:** Newcastle–Ottawa Scale (NOS) scores of the included cohort studies.

Study	Publish a Particular Year	Selection	Comparability	Outcome	Total Points
Kong	2022	★★★★	★★	★★★	Nine stars
Koch	2022	★★★★	★★	★★	Eight stars
Zhang	2026	★★★★	★★	★★	Seven stars
He	2026	★★★	★★	★★	Eight stars
Zhao	2025	★★★★	★★	★★	Eight stars
Stachon	2019	★★★★	★★	★★★	Nine stars
Alharbi	2020	★★★★	★★	★★	Eight stars
Zhou	2023	★★★★	★★	★★★	Nine stars
Mentias	2023	★★★	★★	★★	Seven stars

## Data Availability

Data and materials are available.

## Supplementary Materials

The following supporting information can be downloaded at: https://www.mdpi.com/article/10.3390/jcdd13100486/s1, S1: PRISMA 2020 checklist.
