# Supplementary material for: Clinical Outcomes of Transcatheter Versus Surgical Aortic Valve Replacement in Patients with Pure Native Aortic Regurgitation: A Systematic Review and Meta-Analysis
Source: J Cardiovasc Dev Dis. 2026 Sep 26;13(10):486. doi: 10.3390/jcdd13100486 (PMC13705365; doi:10.3390/jcdd13100486)
Supplement: Supplementary file 1 [file jcdd-13-00486-s001.zip › jcdd-4525849-supplementary.pdf]

Supplementary Material S1: PRISMA 2020 checklist

Item No.	PRISMA 2020 Item Description	Location in Manuscript	Reporting Status	Remarks
Title and Abstract				
1	Title: Identify the report as a systematic review, and include meta-analysis if performed	Title page, manuscript title	Fully reported	The title explicitly states "A Systematic Review and Meta-Analysis"
2	Abstract: Provide a structured abstract containing background, objectives, data sources, eligibility criteria, study selection, data extraction, synthesis methods, results, limitations, conclusions and registration number	Abstract section	Fully reported	The structured abstract includes background, methods, results and conclusions; PROSPERO registration number CRD420261411339 is provided
Introduction				
3	Background: Describe the rationale and context for the review	Section 1. Introduction	Fully reported	Describes disease background of pure native aortic regurgitation, clinical status of SAVR and TAVR, and inconsistencies among existing studies
4	Objectives: State the review question incorporating PICO elements (Population, Intervention, Comparator, Outcomes)	Final paragraph of Section 1. Introduction	Fully reported	Population: adult patients with pure native aortic regurgitation; Intervention: TAVR; Comparator: SAVR; Outcomes: length of hospital stay, perioperative complications, valve condition, all-cause mortality, cardiovascular mortality, new-onset atrial fibrillation, new permanent pacemaker implantation
Methods				

5	Registration: Provide review registry name and registration number; state reasons if not registered	Section 2. Data Sources	Fully reported	PROSPERO registration: CRD420261411339; the review protocol was completed prior to literature screening
6	Protocol: Indicate whether a pre-specified review protocol exists and its accessibility	Section 2. Data Sources	Fully reported	Statement: “The review protocol was written prior to literature screening.”
7	Information sources: List all databases searched, search date, and additional supplementary search approaches (e.g. reference list checking)	Section 2. Data Sources	Fully reported	Databases: Web of Science, PubMed, EMBASE, Cochrane Library; last search date: July 26, 2026; manual reference screening was performed
8	Search strategy: Present full search strategy for at least one database, including search terms and Boolean operators	Table 1. Search strategy for PUBMED	Fully reported	Complete PubMed search strategy is provided in Table 1
9	Eligibility criteria: Specify inclusion and exclusion criteria based on PICO, as well as study design restrictions	Section 2.1 Eligibility Criteria	Fully reported	Inclusion criteria: adult patients, reporting in-hospital and 30-day post-discharge outcomes; study types including RCTs, cohort and retrospective studies. Exclusion criteria: animal studies, reviews, studies without extractable outcome data, participants concomitant with aortic stenosis
10	Study selection: Describe screening process (title-abstract screening, full-text	Section 2.2 Selection of Studies, Data Extraction, and	Fully reported	Two reviewers independently performed study screening; disagreements were resolved via consultation with a third reviewer; PRISMA flow

	assessment), independent dual screening and dispute resolution	Management		diagram (Fig 1) is mentioned in text
11	Data extraction: Report data extraction procedures, dual-extraction, dispute resolution, and list of extracted variables	Section 2.2 Selection of Studies, Data Extraction, and Management	Fully reported	Extracted items: study characteristics, patient baseline features, in-hospital and 30-day outcomes. Two reviewers independently extracted data; third reviewer mediated disagreements
12	Data items: Define all outcomes and specify time-points for outcome assessment	Abstract, Section 2.2, Section 3.3 Outcome Measures	Fully reported	Primary outcomes: length of hospital stay, perioperative complications, valve condition. Secondary outcomes: all-cause mortality, cardiovascular mortality, new-onset atrial fibrillation, new permanent pacemaker implantation. Time-points: in-hospital and 30-day post-discharge
13	Risk of bias in individual studies: Describe tool used to assess risk of bias / methodological quality of included studies	Section 2.3 Certainty of Evidence	Fully reported	Newcastle-Ottawa Scale (NOS, 9-star scale) was applied for cohort study quality assessment; assessment results are presented in Table 5
14	Effect measures: Specify effect sizes used for meta-analysis (e.g. OR, MD)	Section 2.4 Data Synthesis and Analysis	Fully reported	Odds ratios (OR) with 95% confidence intervals for dichotomous outcomes; mean difference (MD) with 95% confidence intervals for continuous outcomes
15	Synthesis methods: Describe heterogeneity assessment, choice of statistical model (random-effect / fixed-effect),	Section 2.4 Data Synthesis and Analysis	Fully reported	Cochran's Q statistic and I^2 statistic for heterogeneity testing; random-effect model for $I^2 \geq 50\%$, fixed-effect model for $I^2 < 50\%$; analyses performed in RevMan 5.4 software

	pooling approach			
16	Additional analyses: Report sensitivity analyses, subgroup analyses pre-specified in protocol	Section 2.4 Data Synthesis and Analysis; Section 3.3 Outcome Measures	Fully reported	Leave-one-out sensitivity analysis; funnel-plot for publication bias evaluation; subgroup analyses comparing all-cause versus cardiovascular mortality and valve-related endpoints
17	Certainty of evidence: Describe methods used to assess certainty of body of evidence	Section 2.3 Certainty of Evidence	Fully reported	Study quality was evaluated via NOS scale
Results				
18	Study selection: Report number of records identified, excluded at screening, full-text assessed and final included studies; present PRISMA flow diagram (Fig 1)	Section 3.1 Characteristics of the Included Studies	Fully reported	1150 initial records retrieved; 9 studies finally included; PRISMA flow diagram Fig 1 presented in manuscript
19	Study characteristics: Tabulate key features of each included study: study design, sample size, participants, interventions, comparators, outcomes, follow-up	Table 2, Table 3, Table 4	Fully reported	Table 2: study design, sample size, TAVR valve systems and procedural access; Table 3: patient baseline clinical characteristics; Table 4: pre-operative echocardiographic characteristics
20	Excluded studies: List full-text articles excluded and reasons for exclusion	Not separately tabulated in manuscript	Fully reported	(1) Animal studies, in vitro studies, reviews, meta-analyses, duplicate publications, editorials, comments, abstracts of conferences without full text, or qualitative research; (2) Studies that do not have available or cannot extract data on in-hospital outcomes and post-

				discharge 30-day outcomes.; (3) The baseline population includes participants who have AS.
21	Risk of bias in studies: Summarize risk-of-bias / quality assessment results for each included study	Table 5 Newcastle–Ottawa Scale(NOS) scores of the included cohort studies	Fully reported	NOS star ratings for all nine included studies are summarized in Table 5
22	Results of individual studies: For each outcome, report effect estimates and confidence intervals for each primary study	Section 3.3 Outcome Measures; Forest plots Fig2-Fig7	Fully reported	Forest plots generated by RevMan display individual-study effect sizes and corresponding 95% confidence intervals
23	Synthesis of results: Report pooled meta-analysis results, heterogeneity statistics for all pre-specified primary and secondary outcomes	Section 3.3 Outcome Measures	Fully reported	All primary and secondary outcomes are reported with pooled effect size, 95%CI, P-value and I^2 heterogeneity index
24	Additional analyses: Present results from sensitivity and subgroup analyses	Section 3.3 Outcome Measures, Discussion section	Fully reported	Results of leave-one-out sensitivity analysis and pre-defined subgroup comparisons are fully described
Discussion				
25	Summary of evidence: Synthesize main findings and discuss certainty of evidence	Section 4. Discussion	Fully reported	Summarizes benefits and drawbacks of TAVR compared with SAVR; interprets findings in context of study quality and anatomical-device-related factors
26	Limitations: Address limitations of the systematic review and bias risks from	Section 5. Limitations of this Study	Fully reported	Four main limitations are explicitly listed: retrospective observational nature of included studies, between-study heterogeneity, short follow-up duration, limited sample

	included primary studies			size for several outcome endpoints
27	Conclusions: Interpret findings for clinical practice and suggest directions for future research	Section 6. Conclusions; end of Discussion	Fully reported	Clinical implications are discussed; need for prospective multicenter randomized controlled trials is highlighted
